# Impact of hBN Content on the Tribological Behavior and Thermal Diffusivity of HVOF-Sprayed Cr_3_C_2_-NiCr Coatings

**DOI:** 10.3390/ma17225470

**Published:** 2024-11-09

**Authors:** Julian Eßler, Dino Woelk, Ion-Dragos Utu, Gabriela Marginean

**Affiliations:** 1 Materials and Manufacturing Engineering Department, Faculty of Mechanical Engineering, Politehnica University of Timișoara, Bvd. Mihai Viteazu nr. 1, 300222 Timișoara, Romania; julian.essler@student.upt.ro (J.E.); dino-horst.woelk@student.upt.ro (D.W.); dragos.utu@upt.ro (I.-D.U.); 2 Institute of Mechanical Engineering, Westphalian University of Applied Sciences, Neidenburgerstr. 43, 45897 Gelsenkirchen, Germany

**Keywords:** boron nitride composite coatings, HVOF, Cr_3_C_2_-NiCr, sliding wear, thermal diffusivity

## Abstract

Considering the significant health risks posed by hard chrome plating during its application, thermally sprayed Cr_3_C_2_-NiCr cermet coatings represent a suitable alternative. Incorporating hexagonal boron nitride (hBN) as a dry lubricant into the feedstock powder can further enhance wear resistance and thermal conductivity, crucial for preventing premature failure caused by inadequate lubrication. In this study, the mass fraction of hBN was varied between 0 and 15 wt.% to assess its influence on the tribological performance of the coatings using pin-on-disk tests. The coating’s hardness was measured via the Vickers method, and its cracking tendency at the coating/substrate interface was evaluated. Scanning electron microscopy (SEM) and X-ray diffraction (XRD) were employed to analyze the microstructure and phase composition, while thermal diffusivity was determined using the laser flash method. The findings revealed that the inclusion of hBN, at concentrations of up to 10 wt.%, leads to an improvement in thermal diffusivity and a reduction in the coefficient of friction. However, exceeding this threshold leads to a decrease in hardness and increased crack formation tendency, highlighting the trade-off between frictional and mechanical properties.

## 1. Introduction

Hard chrome coatings have been used in industry for numerous applications, for components that have been damaged by increased wear, severe corrosion or high temperatures. However, given the adverse impacts of these coatings on health and the environment throughout their manufacturing process, it has become essential to seek an alternative solution [1,2]. Carbide-based cermet coatings, which contain a relatively soft metallic matrix (Co, Cr or Ni) and a hard material component (carbides), are often used as an alternative [2,3,4,5]. These coatings exhibit similar or even better physico-chemical and mechanical properties relative to hard chromium coatings and are mainly utilized to extend the service life of various components [6,7]. The hardness of HVOF (high-velocity oxy-fuel)-sprayed Cr_3_C_2_-NiCr coatings is higher than 900 HV [3,8]. HVOF spraying is often used as a coating process as it offers high particle velocities and lower process temperatures compared to other thermal spraying techniques (e.g., APS) [9,10].

Owing to its two-dimensional structure, hBN (hexagonal boron nitride) exhibits outstanding properties that make it highly suitable as a dry lubricant. Since hBN only decomposes at temperatures above 2700 °C, composite coatings with a certain amount of such dry lubricant demonstrate significantly improved sliding performance even at temperatures between 400 °C and 1000 °C [11,12,13,14,15,16]. Last but not least, this dry lubricant is characterized by excellent thermal conductivity and low density compared to conventional ceramic coatings [17,18,19]. A variety of research findings illustrate that the addition of hBN can markedly improve wear characteristics, applicable at both room temperature and high-temperature conditions (400–800 °C). However, the effect on thermal conductivity is not often addressed [15,20,21]. Some publications deal with the corrosion resistance of hBN-containing coatings, especially in the presence of aggressive chemicals, harmful microorganisms and at high temperatures [22,23].

In the field of suspension flame spraying of yttrium-stabilized zirconium oxide, it has been demonstrated that the volume of particles introduced and the particle size are the most important factors influencing sliding behavior and mechanical properties. As the hBN content increases, the hardness of the coating decreases, but the lubricating particles can more easily enter existing cavities and be incorporated into the matrix. This means that hBN particles can be continuously released [24,25].

Nevertheless, if the particle size or the hBN content is significantly large, the coatings are likely to exhibit increased susceptibility to cracking due to the inhomogeneous stress present within the coating. In the field of YSZ coatings (yttria-stabilized zirconia), particle sizes from 0.3 µm to 4 µm and an hBN mass fraction of 1–5% have been investigated [24]. The hBN particle size utilized can differ significantly according to the intended application. Some tests involved the selection of particle sizes measuring a few micrometers, whereas other tests employed hBN particles with sizes between 20 and 90 μm for the coating process. [11,15,24]. In the area of cold spraying, it was found that the adhesive strength of the coating decreases with a higher proportion of hBN lubricant incorporated, which is presumably due to the poor adhesion between the constituents. In this case, a maximum admixture level of 6% by weight was regarded as technically appropriate, since exceeding this threshold led to the ineffective incorporation of hBN into the coating [21]. With regard to the already mentioned adhesion problems, it was found that the deposition rate decreases with increasing the hBN content [21,26,27]. Various publications demonstrate that the hBN mass fraction should be kept ≤10% [12,13,21,24]. One of the primary issues encountered in the application of coatings is the poor wettability of hBN with numerous coating materials. As a result, the application of an hBN composite coating via thermal spraying is often a difficult task. However, supersonic plasma spraying has been found to improve the cohesive forces of the hBN composite coating compared to other methods [11,28].

A further outcome of the stated issue is that a substantial portion of the hBN particles present in the coatings demonstrates weak adherence or is located on the surface, independent of the coating process used. This situation additionally reflects a lack of cohesion [15,26].

Particular attention is currently directed towards HVOF-sprayed coatings that are based on Cr_3_C_2_ and WC, into which hBN has been incorporated. It was found that the addition of hBN to HVOF-sprayed Cr_3_C_2_ coatings also reduces the coefficient of friction. The investigation further revealed that the embedded hBN was likely released due to the shear forces generated during the wear test, which subsequently caused it to be displaced and pushed outward [15], similar to what was observed in the previously mentioned publications.

To overcome the problem of segregation in the powder mixture, preliminary actions are undertaken in the preparation and feeding of the powder, or alternatively, adjustments are made to the process selection and settings. Grinding with a mill has been shown to be beneficial for mixing the feedstock material that is suitable for safe coating in the process. The investigation results revealed that the integration of hBN particles into the composite coating was accomplished successfully [21].

Ball mills are not only used to comminute raw materials, but also to improve the mechanical properties of fine-grained materials. Classically, ball milling is used to produce a powder mixture of a solid lubricant and a ceramic for thermal spraying [29]. Another approach for the production of the above-mentioned composite coatings is to apply the high-velocity air fuel technique (HVAF) and feed the solid lubricant directly from an hBN suspension, which significantly increases the complexity of the process due to the suspension [20]. In the studies where HVOF was used, it was demonstrated that the cohesive forces can be improved due to the high kinetic energy [30].

The primary objective is to design a multifunctional coating that fulfills the criteria of wear resistance and low friction, in addition to improving thermal performance. This integrated approach will yield a competitive benefit in a range of applications, particularly within the automotive, aerospace and industrial machinery sectors, where these attributes are vital. This study aims to examine the impact of incorporating hBN material on the performance of HVOF-sprayed Cr_3_C_2_-NiCr coatings, specifically in relation to the microstructure, to sliding wear and thermal diffusivity. This offers both theoretical and technical assistance for the utilization of hBN in the domain of thermal spray coatings designed to be wear-resistant and self-lubricating across a broad temperature spectrum. Focusing on these aims, the newly developed coating intends to provide a reliable and efficient alternative to the currently available solutions, effectively addressing the specific demands of industries in need of durable, high-performance materials.

## 2. Materials and Methods

### 2.1. Feedstock Powder and Coating Deposition

A cermet powder from GTV Verschleißschutz GmbH (Luckenbach, Germany) was used for the present investigation (Cr_3_C_2_-NiCr: 80/20) with an average particle size distribution of +45/−15. The hexagonal boron nitride was produced by the company Henze Boron Nitride Products AG, with an average particle size of 10 ± 2 µm. The powders were deposited by means of the HVOF coating technique (K2 coating gun, GTV Verschleißschutz GmbH) at a particle speed of 400 m/s on high-alloy stainless steel (Ø 25 mm, made of 1.4571 with a roughness of Ra 1.6). The powders were mixed directly in the process by varying the feed rate of hBN and Cr_3_C_2_-NiCr. Thus, a composition of 0% up to 15% hBN content was added to the Cr_3_C_2_-NiCr powder in 5% steps. The coating parameters are listed in Table 1. A significant focus of the composition is its performance under dry conditions, where traditional lubricants may fail. The coating thickness of the samples was 220 ± 26 µm after coating and 150 ± 10 µm after grinding the surface (with 80 grit sandpaper). Layer thickness measurements were performed with a micrometer, evaluating the samples in both their uncoated and coated conditions.

The samples were labeled depending on their hBN content. Therefore, the conventional reference coating was labeled 0 hBN, for example. The other designations and the resulting coating composition can be found in Table 2.

The objective of choosing this composition is to develop an alternative coating that provides substantial wear resistance and effective sliding properties, even under dry operating conditions. By incorporating materials known for their hardness and durability, the coating aims to minimize degradation over time, prolonging the lifespan of the components it protects. By achieving low friction coefficients, the coating will not only enhance the efficiency of moving parts but also mitigate heat generation due to friction, thereby further preserving the integrity of the substrate. Efficient heat dissipation is crucial for avoiding overheating, which can compromise the performance and lifespan of mechanical systems. The selected material composition is designed to improve heat transfer, thus optimizing the thermal management of the components. The powder was extracted from the coating system to allow for an assessment of its structure and the distribution of hBN particles using scanning electron microscopy (SEM).

### 2.2. Microscopical Investigation of the Coating Microstructure

The microstructure of the powder mixture and the deposited coatings were examined through scanning electron microscopy (Sigma 300 VP from Carl Zeiss Microscopy Deutschland GmbH, Oberkochen, Germany) in combination with EDX analysis (energy dispersive X-ray, Oxford Instruments, Abingdon, UK). This investigation specifically aimed to detect the presence of hBN particles within the deposited coating. The metallographic preparation for analyzing the microstructure in the cross-section was conducted as follows:

- Grinding at 320 grit, 500 grit, 800 grit and 1200 grit;
- Polishing with 9 µm and 3 µm diamond suspension.

The phase composition of the coating containing 15% hBN was determined through X-ray diffraction analysis conducted with a Philips X’Pert Diffractometer (Panalytical, Almelo, The Netherlands) utilizing Cu Kα radiation.

The Keyence VK-X3000 from Keyence Deutschland GmbH (Neu-Isenburg, Germany) confocal laser scanning microscope (CLSM) was used for microscopic investigation of the region subjected to Brinell indentation to qualify the coating adhesion and tendency to crack formation.

### 2.3. Hardness and Tribological Tests

The hardness measurements were carried out using a KB 250 universal hardness tester from KB Prüftechnik GmbH (Hochdorf-Assenheim, Germany) in accordance with the Vickers method (HV1). The measurements were repeated twelve times to determine the average value. To investigate the tendency to crack formation, Brinell hardness measurements were carried out using the same device. The Brinell indenter had a diameter of 2.5 mm, and the test force was 62.5 kp. For both measuring methods, the time until the maximum force was applied was 5 s, and the exposure time was 15 s. The indentation of the body was made at the interface between the coating and the substrate. The assessment criteria established are as follows: Cracks found within the indentation zone are regarded as harmless, while those that extend outside this area are considered critical. The separation of the coating from the substrate and the formation of a crack network are also classified as critical or deficient. The dry-sliding environmental wear tests were carried out using a pin-on-disk tribometer from CSM Instruments. The parameters for the test were established as follows: a load of 10 N, a linear velocity of 15 cm/s, a wear track radius of 3 mm, and a stopping condition set at 10,000 laps. The wear ball made of 100 Cr6 had a diameter measuring 6 mm.

### 2.4. Thermal Diffusivity

To determine the thermal diffusivity, 1.5 mm thick 1.4571 disks of 12.6 mm diameter were coated (using the same procedure as described in 2.1). Both the front and back surfaces were ground with 80 grit SiC grit. After preparation, the samples had a total height of 1.15 mm (approx. 0.15 mm coating and 1.0 mm substrate).

The LFA 467 HyperFlash from Netsch Gerätebau GmbH (Selb, Germany) was used to measure the thermal diffusivity at room temperature. The test parameters were selected with a voltage of 200 V and a pulse width of 400 µs. Before testing, the samples were sprayed with graphite. Each set of samples was measured five times to determine the average value.

## 3. Results and Discussion

### 3.1. Powder Characterization

**Figure 1:**
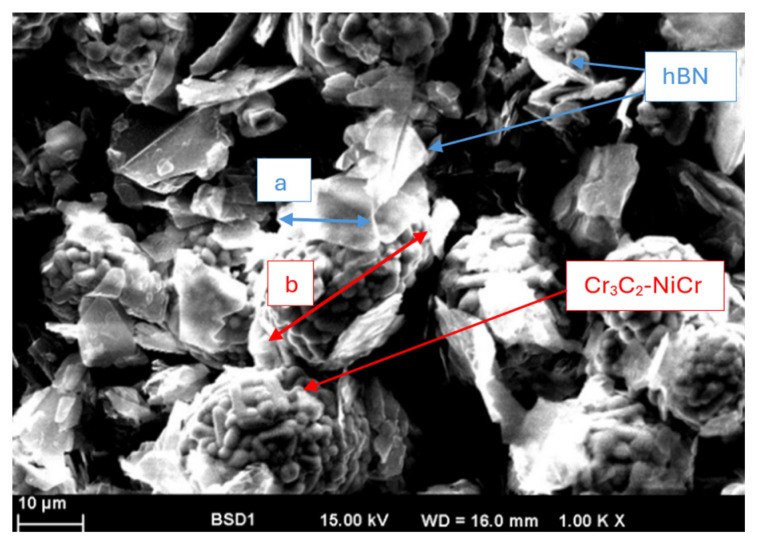
SEM micrograph of the powder mixture containing 10 wt.% hBN.

The directly mixed hBN-Cr_3_C_2_-NiCr powder was taken at the mixing point and investigated by SEM. Figure 1 reveals the way the hBN particles are distributed among the Cr_3_C_2_-NiCr powder mass. The plate-like structure of hBN can be clearly seen here (see the area marked in blue as an example). The size of the particles corresponds approximately to the mentioned batch average value of the hBN powder. The Cr_3_C_2_-NiCr powder shows a spherical shape and is also in the range of the particle size distribution specified by the supplier.

### 3.2. Microstructure

**Figure 2:**
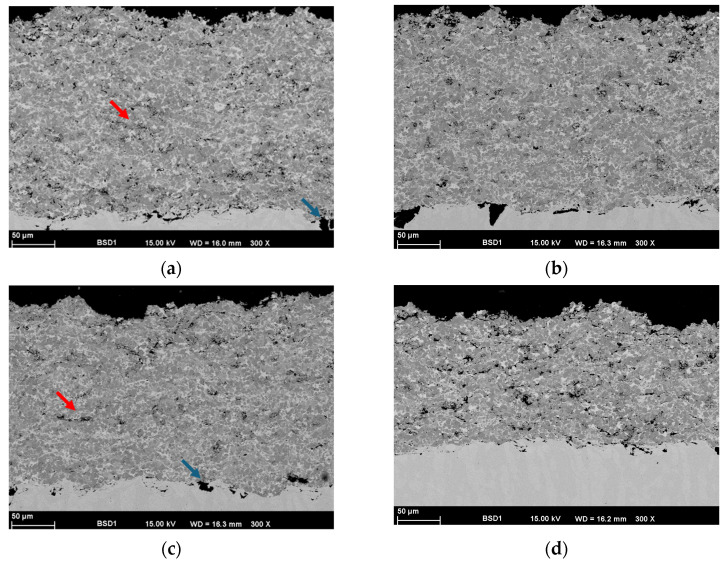
SEM micrographs of the sprayed coatings in cross-section: (**a**) 0 hBN, (**b**) 5 hBN, (**c**) 10 hBN, and (**d**) 15 hBN.

In order to obtain some information concerning the layer quality and at the same time to be able to prove that hBN is embedded in the layer, the samples were investigated in cross-section by means of SEM (Figure 2). Adding hBN into the feedstock powder has not led to any significant change in the coating microstructure. One further remark is worth mentioning, namely, the decrease in the deposition rate for the 15 hBN coating. The data indicates that the coating thickness achieved for 15 hBN is significantly inferior to that of the other samples (see Figure 2a–d). All samples underwent the same number of coating cycles. Therefore, it can be concluded that the deposition rate for this sample was substantially lower (the difference between the density of the two materials is noticeable), and a nearly constant deposition rate can only be expected for hBN concentrations of 10% or less. The results indicate that the deposition rate for this sample was substantially lower compared to other materials, highlighting a significant correlation between the concentration of hexagonal boron nitride (hBN) and the resulting deposition dynamics. The significant variation in density among the materials indicates that an increase in hBN concentration beyond 10% could influence the overall density of the deposited material, potentially impacting the flowability and dispersion of the particles throughout the deposition process. This threshold indicates that exceeding this concentration may lead to variability in the deposition process, potentially resulting in inconsistent coating thickness and performance.

Higher porosity was detected at various points, as demonstrated by the red markings (see the example markings in Figure 2a,c). The particles identified at the interface originate from the blasting process (blue markings) and are identified as Al_2_O_3_ (blasting residues).

**Figure 3:**
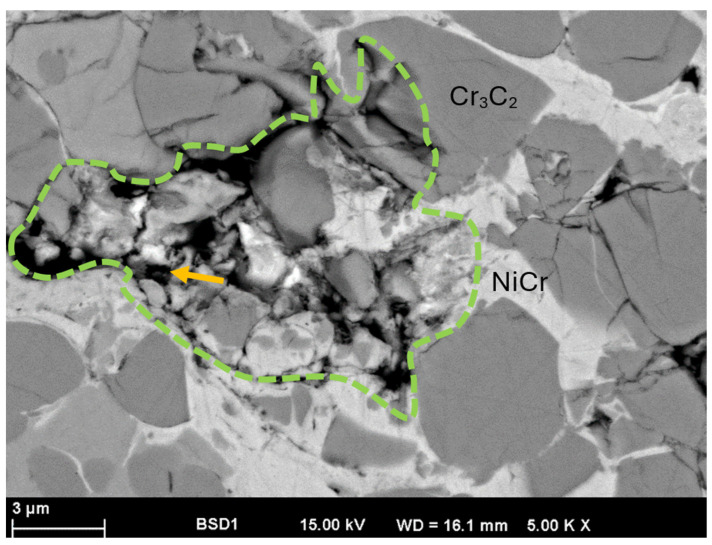
SEM micrograph of the 15 hBN coating in cross-section.

A detailed examination of the coating at higher magnification has provided significant insights into its microstructural properties, especially in differentiating between regions that contain hexagonal boron nitride (hBN) particles and those that display increased porosity. This examination is essential for comprehending the impact of these characteristics on the coating’s overall performance (Figure 3). The presence of hBN particles within the coating is significant, as these particles contribute to essential properties such as wear resistance and thermal conductivity. Porous regions may compromise the mechanical strength of the coating and also alter the thermal conductivity by hindering the effective heat dissipation, leading to localized overheating during operation.

Figure 3 also illustrates the structure and appearance of Cr_3_C_2_ and NiCr. The lighter components of Cr_3_C_2_ are noticeably darker in the BSE mode compared to NiCr.

**Figure 4:**
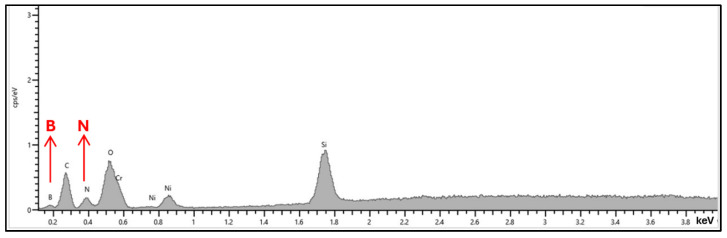
EDX spectrum of an exemplary measuring point marked on sample 15 hBN.

The hBN particles are clearly darker in comparison with the other components of the composite coating. The green outlined area is an example of a possible pull-out of hBN particles during the metallographic sample preparation; otherwise, a larger amount of hBN might have been observed. The size of the break-out site matches the average size of the hBN particles used. The yellow marking in Figure 3 suggests the presence of potential hBN particles, and consequently, EDX measurements were conducted at these specific locations. Figure 4 illustrates the EDX analysis conducted at the point marked by the yellow arrow in that figure. The resulting EDX spectrum confirms the detection of boron (B) and nitrogen (N). The disintegration of hBN particles during the preparation phase serves as evidence of a limited cohesion level. This aspect is also examined in more detail in the tribological investigation.

**Figure 5:**
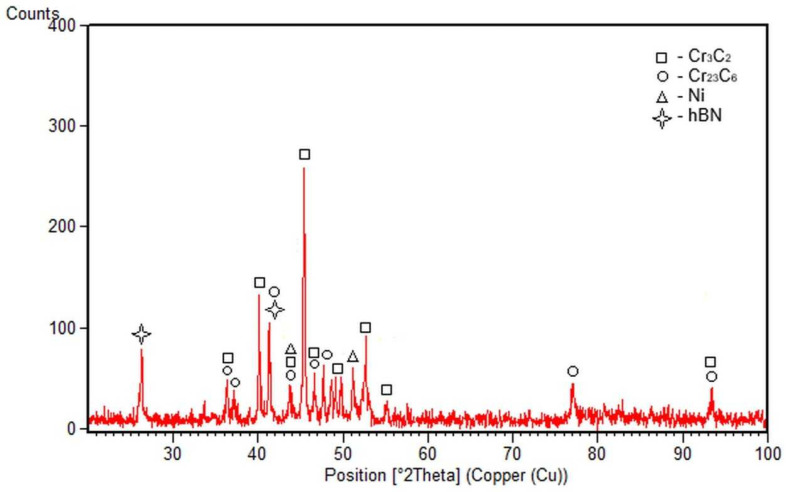
XRD pattern of the composite coating with 15% hBN.

Figure 5 depicts the XRD pattern, which reveals the phase analysis of the composite coating with 15% hBN. The identified phases correlate very well with the results provided by EDX analysis.

### 3.3. Hardness and Tendency to Crack Formation

**Figure 6:**
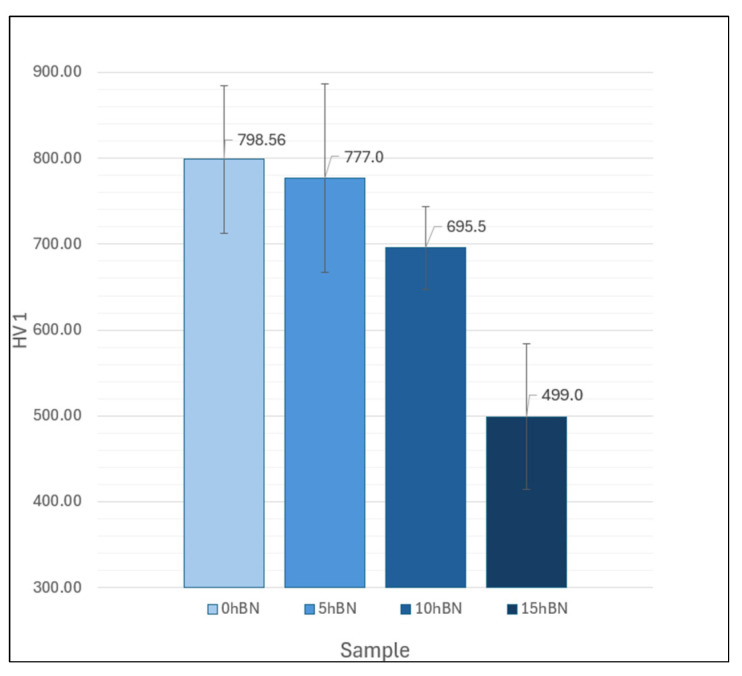
Hardness values of the investigated composite coatings.

The results of the Vickers hardness measurement presented in Figure 6 demonstrate that the hardness of the coating decreases with increasing hBN content. This could be justified by the fact that hBN is softer and less stable in comparison with the cermet particles. This means that the resistance to penetration by a body is mainly determined by the properties of the Cr_3_C_2_-NiCr material. Since less cermet material is present with increasing hBN content, the hardness consequently decreases. The explanation for hBN’s limited contribution to hardness lies in the lack of chemical bonding between hBN and Cr_3_C_2_-NiCr. Effective bonding is crucial for the mechanical reinforcement of composite materials. Without adequate interfacial bonding, hBN particles fail to effectively transmit loads or contribute to the reinforcement of the cermet structure. A more in-depth discussion of this matter will be presented in Section 3.4.

**Figure 7:**
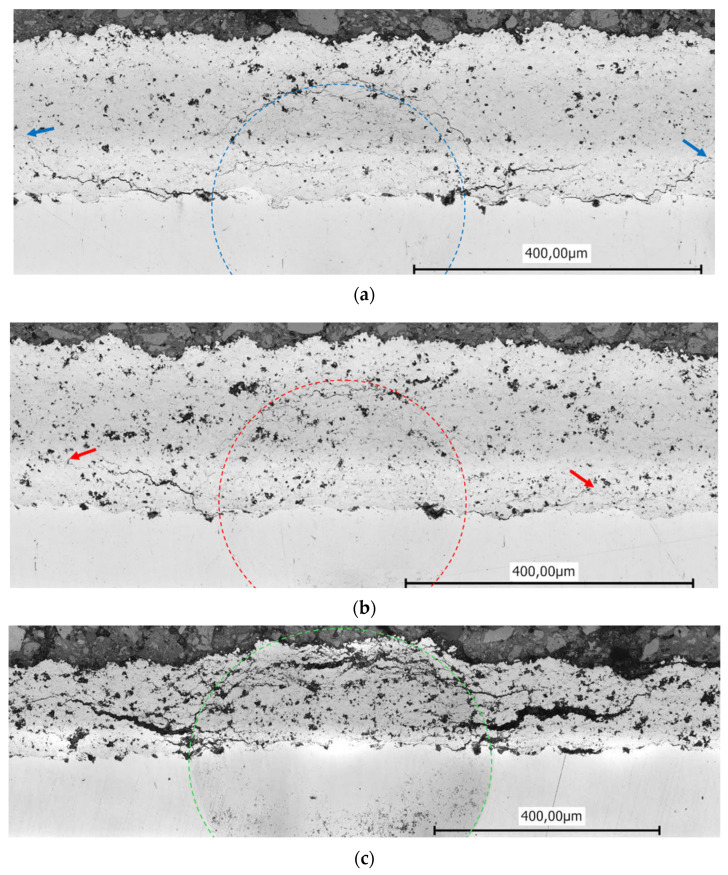
CLSM images of the Brinell indentation area: (**a**) 0 hBN; (**b**) 10 hBN; and (**c**) 15 hBN.

In addition to the hardness measurement, the tendency to crack formation was also tested using the Brinell method. The results from the Brinell hardness test indicate that the presence of hBN influences crack morphology and propagation, revealing important implications for the durability of the coating. The findings demonstrate that with the addition of 10% hBN, less pronounced and shorter cracks that go beyond the indentation area of the sphere (marked by the dashed lines) appeared, see Figure 7a,b. The cracks start at the interface between the coating and the substrate, then take an elliptical course towards the surface and end at the points marked by arrows within the layer. This observation suggests that this boundary plays a critical role in the distribution of stress during indentation. The fact that cracks follow this path highlights the interaction between the mechanical properties of the coating and its adhesion to the substrate.

There are even finer cracks around the indentation areas, but these are also more pronounced in the case of the reference sample (0 hBN) than that identified for sample 10 hBN. The 15 hBN coating exhibited the worst behavior, showing the larges crack progress in the coating. In this case, many cracks developed beyond the indentation limits and even reached the surface. The obtained results correlated very well with the measured hardness, giving an indication for the coating stability against crack initiation and propagation. Up to a concentration of 10% hBN, the hardness decreases moderately, allowing the layer to maintain sufficient resistance against the penetrating body, resulting in relatively small displacements. The introduced hBN, which is loosely encapsulated within the layer, contributes to elasticity. However, once the hBN content reaches 15%, Cr_3_C_2_-NiCr becomes a coating system with a significantly diminished ability to withstand loads. The lack of bonding with hBN results in the formation of cracks, which approach delamination, due to the nearly unrestricted penetration of the Brinell body under the selected testing conditions.

The observed reduction in crack formation due to the presence of hBN suggests that the coating may offer superior durability and enhanced resistance to mechanical stress-related failures. The incorporation of hBN improves the elasticity of the Cr_3_C_2_-NiCr coating at lower concentrations; however, when the concentration surpasses 15%, there is a significant decline in load-bearing capacity attributed to inadequate bonding and increase in crack development. It is crucial to carefully evaluate hBN concentrations to optimize the coating’s performance in challenging applications. This characteristic is particularly pertinent for applications where coatings are exposed to impact or friction, as a decrease in both the length and severity of cracks can lead to improved service life and overall performance.

### 3.4. Friction and Wear Test

**Figure 8:**
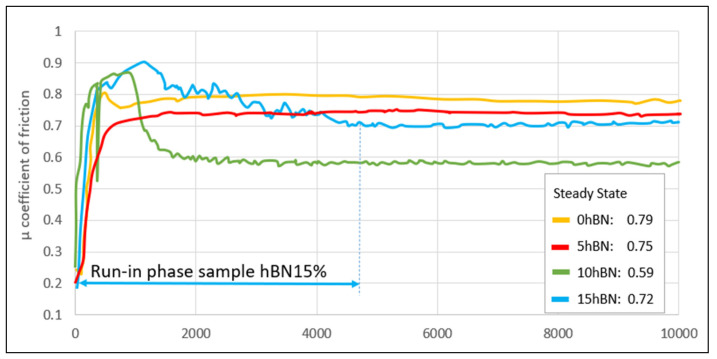
Evolution of the coefficient of friction until it stabilizes at a steady state.

The selection of 100 Cr6 as a counterpart material for the tested coatings is based on several technical applications of such coated surfaces. The results demonstrate that the values for the friction coefficient decrease with increasing the hBN content, as can be seen by comparing the determined steady state values. The coating containing 15 hBN has the longest running-in phase, which is also marked as an example in Figure 8.

The lowest value was registered for the sample 10 hBN, representing an improvement of at least 25% in comparison with the behavior of the reference coating (0 hBN). The different degrees of fluctuations visible in Figure 8, especially for the composite coatings, are in direct correlation with the nature of the tribological film formed on the wear track.

Typically, wear protection layers are designed to have a minimal running-in period and to provide a stable performance with few fluctuations. Variations may occur due to minor spalling, such as those less than 15 µm. The initial phase can be influenced by surface preparation, including cleaning or roughness. Additionally, an effect is anticipated due to the lamellar structure of hBN, which is also reflected in the research findings. The structure of hBN facilitates the sliding of the counterpart over the surface, up to a mass fraction of 10% hBN.

**Figure 9:**
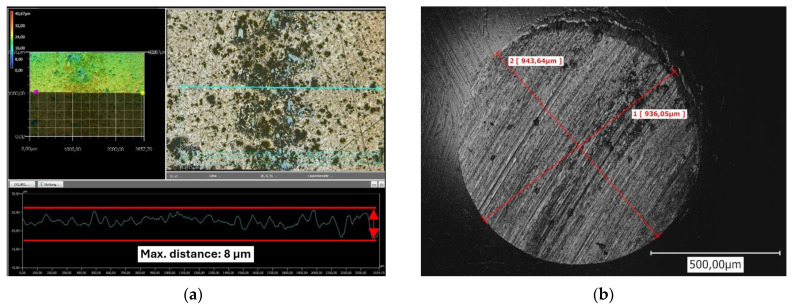
CLSM representation of the wear track profile for the tested 10 hBN coating (**a**) and of the generated contact area on the counter part (**b**).

Figure 9a illustrates a representative 3D scan of a segment of the wear track. The overall scan initially does not display any significant wear volume, as no semicircular shape is evident in the profile line. Consequently, it was not possible to calculate a wear volume for the tested layer. Only in the counterpart (refer to Figure 9b) is a noticeable material removal observed.

**Figure 10:**
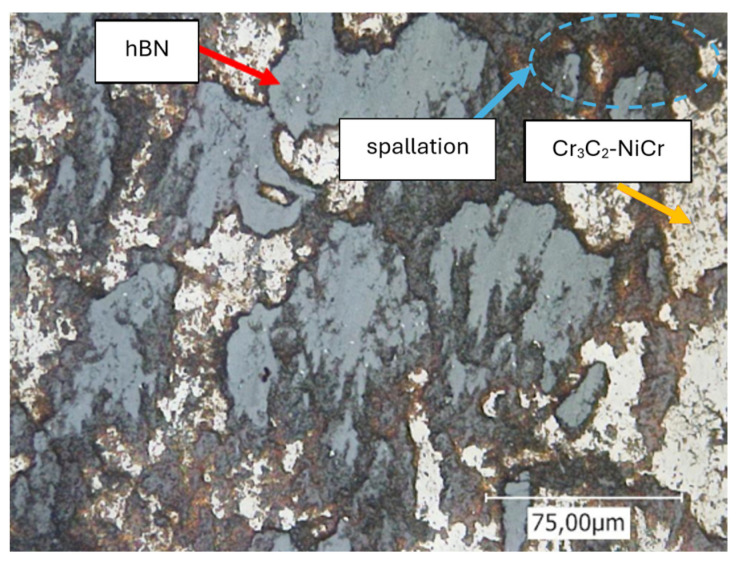
CLSM image illustrating the wear track created on the surface of the 10 hBN coating.

A closer examination of the wear track (refer to Figure 10) reveals that smaller particles have broken away from the upper layer surface. It is well established that during the running-in phase, loosely adhering components of the coating are removed. In the case of cermet coatings, primarily the matrix components, which are significantly softer than the carbides, are deformed or worn away until the carbides are exposed. The wear body then slides over the carbides. Additionally, this particular coating system includes a certain proportion of hBN. Since the hBN particles just physically bond with the Cr_3_C_2_-NiCr layer but are merely surrounded by the cermet material, depending on the amount of hBN introduced, two effects may occur.

When the content of hBN is less than 10%, there exists an adequate cermet layer that ensures the hBN particles remain firmly positioned. The hBN particles that are partially exposed on the surface can gradually release hBN particles, which can then be distributed or lubricated across the surface. The lamellar structure of hBN facilitates the sliding behavior between the wear ball and the coating.

Should the hBN content rise further, similar to the 15 hBN layer, the balance between hBN and Cr_3_C_2_-NiCr becomes detrimental. This situation leads to an inadequate cermet coating, which fails to hold the hBN particles securely. As a result, the hBN particles are dislodged and ejected from the surface, providing no assistance in the development of a lubricating film. The resulting cavities on the surface modify its topography, thereby increasing the frictional resistance.

A maximum hBN content of 10% reveals an improvement in the sliding performance. This suggests that the Cr_3_C_2_-NiCr material adequately maintains the hBN particles in their designated positions, which in turn enhances the sliding properties, as corroborated by the friction coefficient data.

The following Figure 10 illustrates the previously described morphology of the formed tribological film, predominantly consisting of hBN, and is indicated by the red arrow. The area marked in blue highlights the partial detachment of particles, which is caused by lower adhesion forces or an insufficient contact area (envelopment by Cr_3_C_2_-NiCr).

In Figure 10, the proportion of hBN appears significantly larger than that of Cr_3_C_2_-NiCr. This observation can be attributed to the fine distribution of hBN particles (tribofilm formation), which have spread extensively across the surface due to their plate-like hexagonal structure. As a result, this effect may create a false impression that the hBN proportion exceeds the 10% indicated for the sample in Figure 10.

### 3.5. Thermal Diffusivity

**Figure 11:**
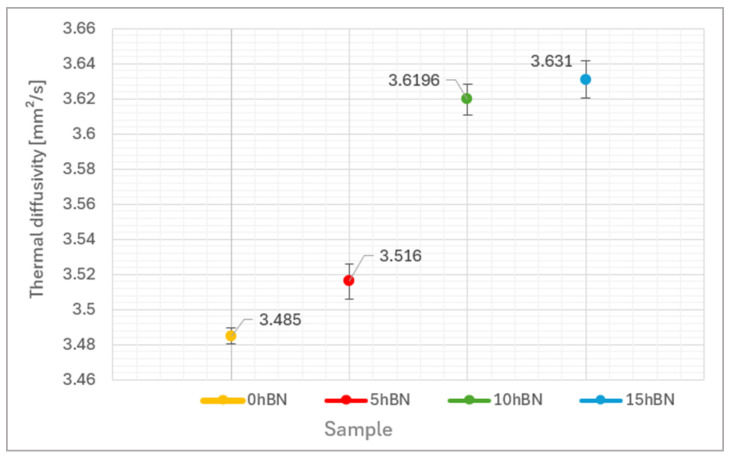
Thermal diffusivity of the samples investigated.

The thermal diffusivity values (Figure 11) measured for the hBN-containing composite coatings in comparison with the reference one, indicate a certain correlation with the above-mentioned wear behavior. Up to 10 wt.% hBN, an increase in thermal diffusivity correlates very well with a decrease of the values for the friction coefficient. The values of the 10 hBN and 15 hBN samples are comparable, which can be attributed to the excessive proportion of hBN that does not sufficiently interact with the Cr_3_C_2_ NiCr material. Since the hBN particles are just enveloped, there may also be localized voids that diminish the beneficial effect of hBN on the thermal diffusivity of the layered system. Consequently, only a slight increase in thermal diffusivity is observed from 10 hBN to 15 hBN. Furthermore, it is noteworthy that the standard deviation among the hBN-containing coatings is relatively similar, yet significantly larger when compared to the reference sample 0 hBN. This is due to the direct mixing method employed in the fabrication of the layered system, which can lead to minor local variations in the hBN content. Additionally, there is a dependence on the orientation of the lamellar hBN particles, which may vary from sample to sample.

## 4. Conclusions

Thermally sprayed Cr_3_C_2_-NiCr coatings with different hBN contents (up to wt. 15%) were successfully deposited by means of the HVOF technique. The influence of adding hBN was investigated with regard to some mechanical properties and correlated with the thermal diffusivity.

The investigation results demonstrated the following:

- An hBN content exceeding 10 wt.% results in a notable reduction in coating hardness and an increased tendency for crack formation. This phenomenon is primarily due to inadequate cohesion between the Cr_3_C_2_-NiCr material and the hBN particles. Therefore, this threshold can be considered optimal for achieving desirable properties in such coatings.
- Comparable observations have been made regarding sliding wear behavior in relation to the measured thermal diffusivity. The friction coefficient values decreased as the hBN content increased, achieving optimal performance at 10 wt.%. However, surpassing this threshold resulted in a deterioration of wear performance, characterized by partial spallation and an increase in the friction coefficient, attributed to weak cohesive strength and inadequate hBN distribution.
- An improvement in thermal diffusivity of up to 10% was observed. However, increasing the hBN content did not lead to any additional enhancement of this physical characteristic.
- An overall assessment of the results obtained indicates a significant potential for the effective application of these composite coatings (up to 10% hBN), which demonstrate enhanced sliding wear resistance and thermal diffusivity.

## Figures and Tables

**Table 1:** Spraying parameters for the coating deposition.

Parameter	Value
Oxygen flow rate [L/min]	695
Spraying distance [mm]	285
Kerosene flow rate [L/min]	0.36
Carrier gas N_2_ [L/min]	11.2
Powder feed rate [g/min]	80

**Table 2:** Name and composition of the coatings.

Sample Name	hBN Feed Rate [g/min]	Cr_3_C_2_-NiCr Feed Rate [g/min]	hBN Feed Rate [%/min]	Cr_3_C_2_-NiCr Feed Rate [%/min]
0 hBN	0	80	0	100
5 hBN	4	76	5	95
10 hBN	8	72	10	90
15 hBN	12	68	15	85

## Data Availability

The data in this study are available on request from the authors. The data are not publicly available because they are part of ongoing studies.
